# High prevalence of picobirnavirus genogroup I in calves without association with diarrhea in a dairy cattle herd, southeast Brazil

**DOI:** 10.1007/s11259-026-11510-y

**Published:** 2026-09-12

**Authors:** Geovana Depieri Yoshitani, Denise Correia Silva, Felipe Augusto de Brito Vargas, Marcos Vinicius de Oliveira, Juliana Torres Tomazi Fritzen, Alice Fernandes Alfieri, Amauri Alcindo Alfieri, Elis Lorenzetti

**Affiliations:** 1 Laboratory of Animal Virology, Department of Preventive Veterinary Medicine, Universidade Estadual de Londrina, PO Box 10011, Celso Garcia Cid Road, PR 445 Km 380, Londrina, Paraná 86057-970 Brazil; 2 Graduate Program in Animal Health and Production, Department of Agrarian Sciences, Universidade Pitágoras Unopar Anhanguera, Arapongas, Paraná 86702-670 Brazil; 3 Multi-User Animal Health Laboratory, Molecular Biology Unit, Department of Preventive Veterinary Medicine, Universidade Estadual de Londrina, PO Box 10011, Celso Garcia Cid Road, PR 445 Km 380, Londrina, Paraná 86057-970 Brazil; 4 National Institute of Science and Technology of Dairy Production Chain II (INCT-LEITE II), Universidade Estadual de Londrina, PO Box 10011, Celso Garcia Cid Road, PR 445 Km 380, Londrina, Paraná 86057-970 Brazil

**Keywords:** Bovine, Fecal sample, Enteric virus, *Orthopicobirnavirus*, PBV GI

## Abstract

Dairy farming is one of the most widespread agricultural activities in Brazil. Neonatal diarrhea remains a major challenge, contributing significantly to economic and production losses in Brazilian dairy cattle herds. Picobirnavirus (PBV) has been suggested as a potential viral agent associated with calf diarrhea. This study aimed to detect PBV genogroup I (GI) RNA in fecal samples from calves in a closed high-producing dairy herd in São Paulo, southeastern Brazil. A total of 122 fecal samples were collected from 30-day-old calves, including diarrheic (*n* = 16) and non-diarrheic (*n* = 106) animals. Nucleic acids were extracted and analyzed using reverse transcription-polymerase chain reaction (RT-PCR). PBV GI was detected in 54.1% (66/122) of the samples, with the majority in non-diarrheic calves (87.9%; 58/66). The detection rate was 50.0% (8/16) in diarrheic calves and 54.7% (58/106) in non-diarrheic calves, with no significant association between PBV GI presence and fecal consistency (*p* = 0.72). Two RT-PCR amplicons from fecal samples collected on different days were sequenced to confirm detection of PBV GI; however, only one nucleotide (nt) sequence presented sufficient quality for analysis. Phylogenetic analysis revealed that this sequence clustered within PBV GI and shared the highest nt identity with a raw sewage sequence (93.4%), two human sequences (87.8–89.8%), and a monkey sequence (88.8%). These results confirm the circulation of PBV GI in a dairy herd and suggest that its presence is not associated with calf diarrhea. Further studies are needed to clarify the epidemiological and biological significance of PBV GI in cattle, as well as its impact on animal health and production.

## Introduction

Neonatal diarrhea is considered a multietiological and multifactorial syndrome that results from the interaction between calves and infectious agents such as viruses, protozoa, and bacteria, and its occurrence may also be influenced by factors including management practices, environmental conditions, nutrition, and the animal’s immune status (Bendali et al. 1999; Cho et al. 2013). Furthermore, since passive immunity in newborn calves initially depends on the transfer of immunoglobulins through colostrum, calves are particularly susceptible to infections during the first weeks of age (Windeyer et al. 2014).

Neonatal diarrhea is frequently associated with coinfections involving multiple pathogens. Among the viral agents implicated in its etiology, rotavirus A (RVA), bovine coronavirus (BCoV), and bovine viral diarrhea virus (BVDV) are recognized as major pathogens, whereas picobirnavirus (PBV) represents a potential contributor (Cho et al. 2013; Malik et al. 2014).

PBVs, in the *Picobirnaviridae* family and *Orthopicobirnavirus* genus, are currently classified into three species: *Orthopicobirnavirus beihaiense*, *Orthopicobirnavirus equi*, and *Orthopicobirnavirus hominis* (ICTV 2025). In humans, PBVs are small, spherical, non-enveloped, and isometric particles with a triacontahedron capsid, measuring 33 to 37 nm in diameter (Delmas et al. 2019). Their genome consists of two linear double-stranded RNA (*ds*RNA) segments. Segment 1 has three open reading frames (ORF1, ORF2, and ORF3); ORF3 encodes a capsid protein precursor, while the functions of ORF1 and ORF2 are unclear. Segment 2 encodes the RNA-dependent RNA polymerase (RdRp), which is essential for viral replication (Delmas et al. 2019).

In addition, PBVs can be classified into three genogroups (GI, GII, and GIII) based on the genomic similarity or divergence in the amino acid sequence of segment 2 (Delmas et al. 2019). Genogroups I and II have been associated with infections in vertebrate hosts, while genogroup III has been identified in invertebrates (Delmas et al. 2019).

PBVs have been detected in a wide range of hosts, including mammals, reptiles, birds (Malik et al. 2014), and invertebrates (Shi et al. 2016). In cattle, PBV has been identified in both diarrheic and non-diarrheic fecal samples in studies conducted in Brazil (Buzinaro et al. 2003; Takiuchi et al. 2016; Chagas et al. 2024; Navarro et al. 2018) and worldwide (Malik et al. 2011; Woo et al. 2019; Nazaktabar 2021; Atasoy et al. 2022).

Regarding the pathogenesis of PBV, it is known that infected hosts may exhibit gastrointestinal alterations; however, current knowledge about the virus’s pathogenic mechanisms and other characteristics remains limited (Rosen et al. 2000). In Brazil, only a few studies have reported the detection of PBV in bovine fecal samples using polyacrylamide gel electrophoresis (PAGE) and/or reverse transcription-polymerase chain reaction (RT-PCR) techniques (Buzinaro et al. 2003; Takiuchi et al. 2016; Chagas et al. 2024; Navarro et al. 2018).

Therefore, further investigations and the application of laboratory diagnostic methods for viral agents such as PBV are essential to expand scientific knowledge about this virus, particularly its role in the neonatal diarrhea complex in calves and its circulation in cattle herds in Brazil. Additionally, such studies contribute to the development of effective control, prophylaxis, and treatment strategies, helping to minimize or prevent the negative impacts of the disease on dairy production.

This study aimed to perform molecular detection of PBV GI in dairy heifer calves with and without diarrhea from a closed high-production dairy herd from the state of São Paulo, Brazil.

## Materials and methods

### Fecal samples

Between March and May 2017, 122 fecal samples were collected from 122 calves aged 30 days, with 16 calves having diarrhea and 106 calves without diarrhea. The samples originated from a closed high-producing dairy cattle herd that averages 37 L of milk/cow/day, located in the State of São Paulo, southeastern Brazil, within the state’s main dairy-producing region. The herd is operated as a closed dairy cattle system with approximately 1,800 Holstein cows in lactation. The cows were managed in a free-stall system under good nutritional and health management practices. The calves were raised in individual pens with elevated floors, and colostrum intake was monitored to ensure adequate consumption by all newborn calves. The fecal consistency scoring, ranging from 0 to 3, was applied to characterize fecal consistency (0, normal; 1, pasty; 2, soft; 3, liquid). Samples scored as 0 or 1 were classified as non-diarrheic, while scores of 2 or 3 were considered diarrheic. The fecal samples are part of a specimen bank maintained by the Laboratory of Animal Virology at the Universidade Estadual de Londrina. All samples were stored at -20 °C until further processing. The study was approved by the Ethics Committee on Animal Use under protocol n. 072/2013, n. 6371.2013.43.

### Nucleic acid extraction

Fecal suspensions (10 to 20% w/v) were prepared by diluting the samples in Tris/Ca^++^ buffer (0.89 mM Tris; 1.5 mM CaCl_2_; pH 7,2). The suspensions were centrifuged at 1,500 x *g* for 10 min, and 500 µL of the supernatant was collected for nucleic acid extraction using phenol/chloroform/isoamyl alcohol (25:24:1) and silica/guanidine isothiocyanate (Alfieri et al. 2006). Nucleic acids were eluted in 50 µL of diethylpyrocarbonate-treated water and stored at -20 °C until further use. Sterile ultrapure water and a fecal sample positive for PBV GI by RT-PCR and genomic sequencing were used as negative and positive controls.

### Reverse transcription-polymerase chain reaction

The nucleic acids extracted from the fecal samples were submitted to RT-PCR assay to amplify a 201 bp fragment of the RdRp gene of PBV GI, using the primers PicoB25 and PicoB43 (Rosen et al. 2000). Aliquots of 5 µL of the amplified products were analyzed by electrophoresis on a 2% agarose gel prepared in Tris-Boric acid-EDTA buffer (89 mM Tris; 89 mM Boric acid; 2 mM EDTA; pH 8.4). The agarose gels were stained with ethidium bromide (0.5 µg/mL), visualized under ultraviolet light, and digitally documented.

### Sequencing and phylogenetic analysis

Two RT-PCR amplicons were selected for purification using the Wizard^®^ SV Gel and PCR Clean-Up System (Promega^®^, Madison, WI, USA) and quantified with a Qubit fluorometer using the Quant-iT dsDNA BR Assay kit (Invitrogen Life Technologies, Eugene, OR, USA). Sequencing was performed on an ABI3500 Genetic Analyzer sequencer (Applied Biosystems, Foster City, CA, USA) using the BigDye Terminator v3.1 Cycle Sequencing kit (Applied Biosystems, Foster City, CA, USA). Sequence quality analysis was performed using the PHRED software and consensus sequences were generated using CAP3 software (https://lbi.cenargen.embrapa.br/phph/). Nucleotide (nt) similarity was evaluated with those deposited in GenBank using the Basic Local Alignment Search Tool (http://blast.ncbi.nlm.nih.gov/Blast.cgi). Sequence alignments were performed using ClustalW in Molecular Evolutionary Genetics Analysis software (MEGA) version 12.1. The phylogenetic tree based on nt sequences was constructed using the Maximum Likelihood method with the Tamura 3-parameter model with Gamma distribution (five categories) and evolutionary invariant sites, also in MEGA version 12.1. Statistical support was assessed by bootstrapping with 1,000 replicates. The nt sequence matrix was performed using Bioedit software version 7.2.5.

### Statistical analysis

The association between PBV GI detection by RT-PCR and fecal consistency (diarrheic vs. non-diarrheic) was assessed using the chi-square (χ²) test to compare proportions between groups. A significance level of 5% (*p* < 0.05) was adopted, and 95% confidence intervals (95% CI) were calculated for differences between proportions. Descriptive data are presented as absolute numbers and percentages (%). All statistical analyses were performed using Minitab^Ⓡ^ version 22.1.

## Results

Among the diarrheic fecal samples (*n* = 16), 8 (50.0%) tested positive and 8 (50.0%) negative for PBV GI. In the non-diarrheic group (*n* = 106), 58 (54.7%) were positive, and 48 (45.3%) were negative. Overall, 66 (54.1%) fecal samples were positive, and 56 (45.9%) were negative for PBV GI. No statistically significant association was observed between PBV GI detection and fecal consistency (*p* = 0.72), as shown in Table 1.

**Table 1:** **–** Results of reverse transcription-polymerase chain reaction assay for picobirnavirus genogroup I detection according to the consistency of the fecal samples collected from dairy calves in São Paulo, southeastern Brazil, 2017

Fecal consistency	RT-PCR PBV GI (%)		
	Positive	Negative	Total
Diarrheic	8 (50.0)	8 (50.0)	16
Non-diarrheic	58 (54.7)	48 (45.3)	106
Total	66 (54.1)	56 (45.9)	122 (100)

*p* = 0.72

RT-PCR: reverse transcription-polymerase chain reaction

PBV GI: picobirnavirus genogroup I

**Fig. 1:**
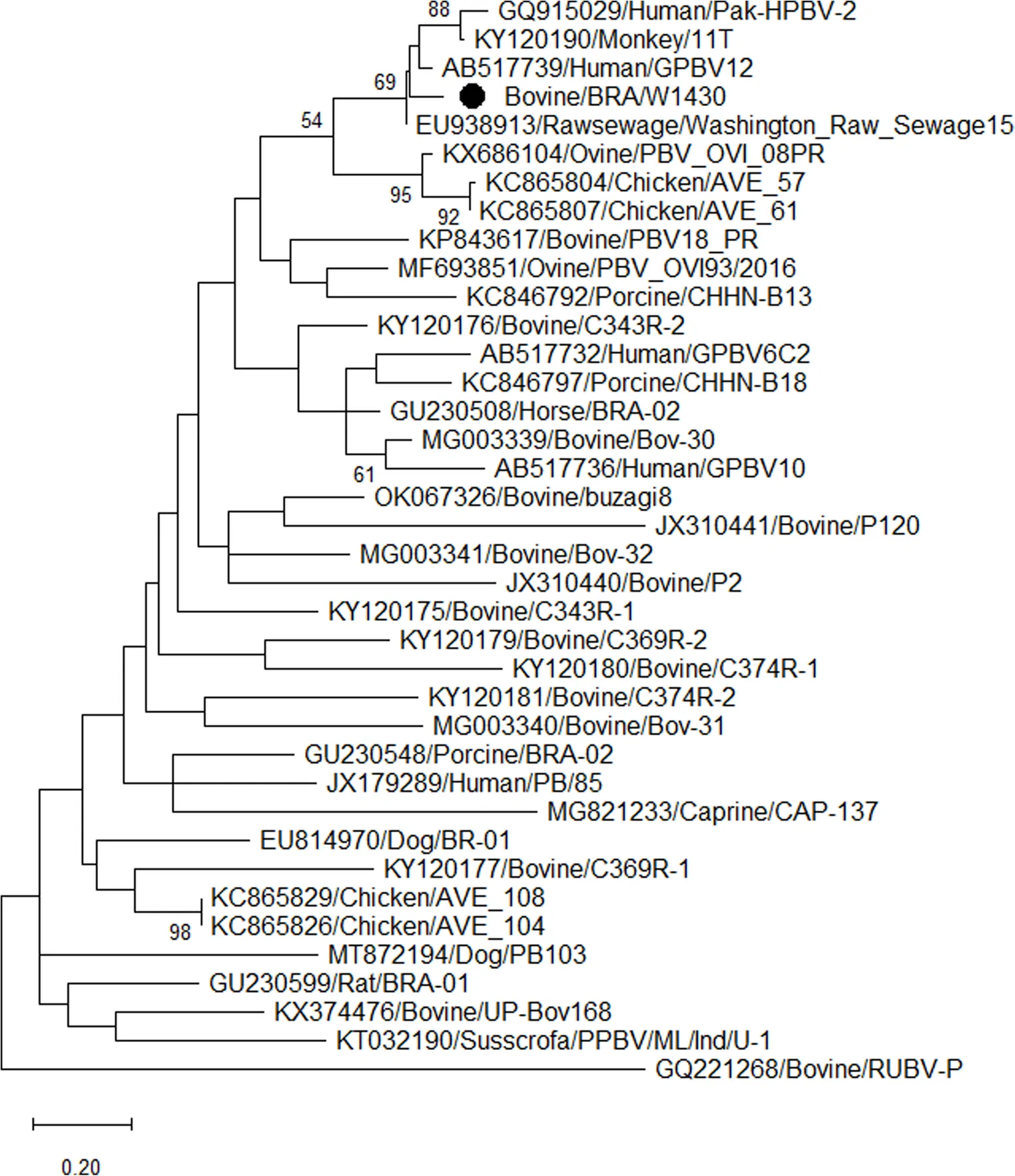
Phylogenetic analysis based on partial nucleotide (nt) sequences (198 nt; positions 757–954) of the RdRp gene (segment 2) of picobirnavirus (PBV). The bovine PBV sequence obtained in the present study is indicated by a black circle. The phylogenetic tree was constructed using the Maximum Likelihood method with the Tamura 3-parameter nt substitution model and gamma distribution (five categories) with evolutionary invariant sites. Statistical support was assessed by bootstrapping with 1,000 replicates. The scale bar represents nt substitutions per site

The specificity of the RT-PCR amplicons was confirmed through sequencing and nt sequences analysis, comparing the results with Sanger sequences available in public databases. Two representative amplicons, obtained from fecal samples collected on different days, with better intensity bands in agarose gel electrophoresis, were selected for sequencing. However, despite repeated sequencing attempts, only one sequence had sufficient nucleotide quality for phylogenetic analysis (Fig. 1). This sequence named W1430 was deposited in GenBank under the accession number PZ490738.

The PBV GI sequence obtained in this study clustered with other PBV GI sequences in the phylogenetic tree and exhibited high nt identity with sequences described in raw sewage from United States of America (93.4% of identity with Washington_Raw_Sewage15, GenBank accession number: EU938913), human from India (89.8% of identity with GPBV12, GenBank accession number: AB517739) and the United States of America (87.8% of identity with Pak-HPBV-2, GenBank accession number: GQ915029), and monkey from China (88.8% of identity with 11T, GenBank accession number: KY120190). Fifteen bovine PBV sequences were included in the analysis, showing 64.9 to 72.5% of nt identity to the bovine PBV sequence from this study, including 71% of nt identity to the Brazilian sequence PBV18_PR (GenBank accession number: KP843617).

## Discussion

In the analysis of PBV GI by RT-PCR assay, this study demonstrated a detection rate of 54.1%, which is higher than the 23.4% (18/77) and 13% (3/23) reported in Brazil by Navarro et al. (2018) and Chagas et al. (2024), respectively. In contrast, Takiuchi et al. (2016), also working with fecal samples from dairy calves in Brazil, reported a higher positivity rate of 62.5% (15/24).

Regarding fecal consistency and the detection of the PBV GI RdRp gene fragment by RT-PCR technique, 50% (8/16) of the positive samples in the present study were from diarrheic calves, an intermediate proportion compared to previous Brazilian cross-sectional studies that reported 73.3% (11/15) (Takiuchi et al. 2016) and 27.8% (5/18) (Navarro et al. 2018). In the latter study, animals aged ≤ 6 months and between 6 months and 2 years were evaluated, with most RT-PCR-positive samples (61.1%; 11/18) originating from calves aged ≤ 6 months.

The detection of PBV in 54.1% (66/122) of the fecal samples confirmed the circulation of PBV GI within the high-production dairy cattle herd studied. In contrast, 45.9% (56/122) of the fecal samples were negative, which may indicate either the absence of PBV infection or the possibility of an early-stage infection in which viral shedding in feces has not yet occurred. Additionally, eight negative samples were diarrheic, suggesting other enteric pathogens (viruses, bacteria, or protozoa) or non-infectious factors, such as management or nutrition.

The detection of PBV in non-diarrheic (58/106; 54.7%) samples suggests viral circulation in asymptomatic animals, as previously reported in the literature (Malik et al. 2018). The role of PBV as a primary causative agent of diarrhea remains unclear, as it has been detected in both diarrheic and non-diarrheic fecal samples (Buzinaro et al. 2003; Malik et al. 2011; Takiuchi et al. 2016; Navarro et al. 2018), including in the present study. A definitive causal link between PBV and diarrhea could not be established in the evaluated calves, as the diarrheic samples tested negative exclusively for RVA and BCoV (data not shown).

Regarding the phylogenetic analysis performed in this study, in comparison with other studies from Brazil, it is noteworthy that one of the bovine PBV sequences analyzed by Navarro et al. (2018) also showed high (75.2%) nt identity with PBV sequences described in human hosts. Similarly, the bovine PBV strain described by Takiuchi et al. (2016) exhibited 81% nt identity with a strain reported in a turkey host. Worldwide, a study realized in Turkey also had some sequences with greater identity to raw sewage sequences, with nt identity percentages ranging from 75.1% to 76.6% (Atasoy et al. 2022). In Iran, PBV nt sequences clustered with humans and monkeys’ strains from different countries (Nazaktabar 2021).

Although the analyzed amplicon corresponds to a fragment of the RdRp gene, which is more conserved among PBV strains, the bovine PBV described in this study is more likely to be a non-bovine PBV strain. These findings reflect the high genetic diversity of this virus, which has also been highlighted in recent studies conducted in countries such as Türkiye and Uganda (Atasoy et al. 2022; Knox et al. 2023).

Other studies have reported the detection of PBV in bovine nasopharyngeal swab samples using molecular techniques (Ng et al. 2015; Woo et al. 2019), suggesting possible tropism of this viral agent for the bovine respiratory tract.

However, there is a scarcity of studies in literature that provide detailed information about the characteristics of the PBV, its role in the development of clinical signs such as diarrhea, and its prevalence in cattle herds. It is essential to expand research about PBV epidemiology and pathogenic potential.

## Conclusion

This study identified the presence of PBV RNA in fecal samples from heifer calves with and without diarrhea, using an RT-PCR assay targeting GI and sequencing of the amplicons. The results indicate the circulation of PBV GI in a high-yield dairy cattle herd from the state of São Paulo, Brazil. Finally, this study demonstrates high prevalence of PBV GI in dairy calves without being associated with neonatal diarrhea.

## Data Availability

The nucleotide sequence obtained in this study was submitted to GenBank under accession number PZ490738.
